# The application of foraging theory to the information searching behaviour of general practitioners

**DOI:** 10.1186/1471-2296-12-90

**Published:** 2011-08-23

**Authors:** Mai Dwairy, Anthony C Dowell, Jean-Claude Stahl

**Affiliations:** 1 Department of Primary Health Care and General Practice, Wellington School of Medicine, University of Otago, New Zealand; 2 Museum of New Zealand Te Papa Tongarewa, Wellington, New Zealand

**Keywords:** Information Foraging, General Practitioners, Evidence - based medicine, Information Seeking Behavior, Questionnaires.

## Abstract

**Background:**

General Practitioners (GPs) employ strategies to identify and retrieve medical evidence for clinical decision making which take workload and time constraints into account. Optimal Foraging Theory (OFT) initially developed to study animal foraging for food is used to explore the information searching behaviour of General Practitioners. This study is the first to apply foraging theory within this context.

Study objectives were:

1. To identify the sequence and steps deployed in identifiying and retrieving evidence for clinical decision making.

2. To utilise Optimal Foraging Theory to assess the effectiveness and efficiency of General Practitioner information searching.

**Methods:**

GPs from the Wellington region of New Zealand were asked to document in a pre-formatted logbook the steps and outcomes of an information search linked to their clinical decision making, and fill in a questionnaire about their personal, practice and information-searching backgrounds.

**Results:**

A total of 115/155 eligible GPs returned a background questionnaire, and 71 completed their information search logbook.

GPs spent an average of 17.7 minutes addressing their search for clinical information. Their preferred information sources were discussions with colleagues (38% of sources) and books (22%). These were the two most profitable information foraging sources (15.9 min and 9.5 min search time per answer, compared to 34.3 minutes in databases). GPs nearly always accessed another source when unsuccessful (95% after 1^st ^source), and frequently when successful (43% after 2^nd ^source). Use of multiple sources accounted for 41% of searches, and increased search success from 70% to 89%.

**Conclusions:**

By consulting in foraging terms the most 'profitable' sources of information (colleagues, books), rapidly switching sources when unsuccessful, and frequently double checking, GPs achieve an efficient trade-off between maximizing search success and information reliability, and minimizing searching time. As predicted by foraging theory, GPs trade time-consuming evidence-based (electronic) information sources for sources with a higher information reward per unit time searched. Evidence-based practice must accommodate these 'real world' foraging pressures, and Internet resources should evolve to deliver information as effectively as traditional methods of information gathering.

## Background

General Practitioners (GPs) provide first contact and continuity with the medical system in many countries, and the way they manage clinical decision-making has a significant impact on both health outcomes and cost effectiveness. Evidence-based medicine (EBM) is currently promoted as the best approach to facilitate the transfer of results from medical research to clinical practice [1]. Use of this evidence has been supported by the development of computerized information retrieval systems and evidence-based resource databases such as Medline and the Cochrane Library [2,3]. Access to medical evidence has been revolutionised by the increasing speed and sophistication of Internet search engines such as Google and Google Scholar [4,5].

General practitioners have supported the promotion of EBM [6,7], but have identified the challenges inherent in applying EBM approaches within the constraints of everyday practice. The primary barriers preventing physicians from answering clinical question were identified in several studies as "lack of time" and "information overload" [8,9].

Understanding information management approaches and constraints in primary care settings is important given the pivotal role of GPs in healthcare. This paper describes a model for understanding GP information searching using Optimal Foraging Theory (OFT), developed to study patterns and strategies of animal foraging [10,11]. Under OFT, the behaviour of an individual forager is broken down into a sequence of discrete behavioural steps (e.g. where to forage, which prey to pursue). At each step, the forager can choose between alternative decisions, and choices may be limited by intrinsic constraints (e.g. foraging skills) and/or extrinsic constraints (e.g. time available, resource distribution). Costs and benefits of alternative foraging decisions are measured using one or a set of currencies (e.g. energy gained per unit time). Central to deriving optimal decisions is the principle of lost opportunity, whereby choosing one activity precludes engaging in an alternative activity which may be more profitable under a given currency and set of constraints.

The two conventional models of OFT are the prey and patch models. Prey models predict that prey types are ranked by profitability (ratio of energy gained to energy spent), and that inclusion of a prey type depends on profitability and encounter rate relative to those of other prey types. Patch models deal with foraging in an environment where resources are clumped (e.g. fruiting trees, schools of fish) and a forager has to allocate its time between foraging within a patch versus searching for a new patch. Patch models generally assume that a forager assesses the quality of a patch by means of the net rate of energy gain. The main prediction of patch models is that a forager should leave a patch when the instantaneous intake rate drops below the average rate in all patches [12].

OFT has been applied in an information-seeking context, whereby information searchers become the foragers, and information items their "preys" [13-15]. Like animal foragers, information seekers have to navigate in a patchy environment in which information items are clumped within discrete patches (e.g. professional colleagues, journals, books, websites, temporary collections constructed by a retrieval programme or search engine). Information foraging analyzes trade-offs in the value of information gained against the costs of performing the activity of human information searching.

Sandstrom [13] argues that because OFT integrates deductive models of evolutionary biology and microeconomics, it lays the groundwork for cost-benefit analyses that can be successfully applied to all human choice-making phenomena, including decisions associated with information behaviour. Pirolli and Card [16] argue that in an information-rich world, the problem is not so much how to collect more information but rather how to optimize the user's time in an attempt to increase relevant information gained per unit time spent. Despite some application to clinical practice, for example the analysis of risky choices made by heroin addicts [17] there has been no published research which applies foraging theory to information management in general practice

The aim of this study was to explore OFT as a tool to understand and model the information seeking behaviour of GPs, and apply it to measure costs (time spent) and benefits of information seeking decisions (finding a satisfactory answer) by GPs.

## Methods

The information foraging behaviour of GPs in the greater Wellington region of New Zealand, was investigated by means of self-completed Clinical Information Search logbooks (i.e. self-completion diaries), combined with background Questionnaires. The research was carried out during 2005; at this time access to the internet was widespread in New Zealand. There was however variability in internet access for General Practitioners at their place of work, and not all would have had access during the consultation. Compared to many other OECD countries at that time, New Zealand had slow broadband speed usage with most internet service providers offering between 256 kbit/s to 1 Mbit/s download speeds. Downloading of information during the consultation was thus relatively limited.

A total of 115 (74.2%) out of 155 eligible registered GPs agreed to participate in the study. The researcher met with each participating GP in an initial face-to-face interview lasting 20-30 minutes in the GP's practice to fill-in their background questionnaire and train them to fill-in their logbook. Logbook questions pertained to an information search, and decisions and rewards within each source accessed. A written reminder was sent to those GPs who did not send back the logs within four to five weeks of the date of the interview; this was followed up with telephone call reminders. Overall, 71 (62% of participants) returned their completed information search logbook.

GPs were given one logbook in which they were asked to document their next information search initiated during routine practice, excluding minor aide memoir checks such as drug dose searches. Clinical Information Search logbooks that were designed, pilot tested and used for this study were based on optimal foraging study protocols, and aimed at providing quantitative data on initial conditions of a search (e.g. information need, time available for the search), and organisation and outcome of an actual information search. GPs were first asked to describe the detailed information question that prompted the search, the constraints faced (importance and urgency of finding the information, maximum time available), and their expectations from the search (estimated time required and likelihood of finding the information). Information needs were classified as simple, simple to complex, or complex (complexity rating 1 to 3 respectively). In the remainder of the logbook, GPs were asked to describe their search behaviour, searching time and success, and decision rules used in each of the information sources sequentially accessed. For each information source accessed, GPs were asked to provide their reasons for choosing this source, their history in searching this source, access time, search time, stopping rules for searching the source and perceived search outcome (Additional file 1, Appendix 1)

The data analysis methods used were: descriptive statistics, chi-square for comparisons of categorical data, and analysis of variance (ANOVA) for comparisons of continuous variables. All tests were with alpha set at 0.05 to determine statistical significance. Results are mostly given as mean ± SD.

Local Ethics Committee approval was obtained for the study.

## Results

### Initial conditions of an information search

Given perceived time constraints in their practice, GPs felt they could allocate up to an average of 15.8 ± 14.3 minutes (median 10, range 1 - 60, n = 70) to address an information need. They anticipated that it would take them 17.3 ± 26.6 min (median 10, range 1-180, n = 69), and rated their chances of success at 71.8% ± 31.8% (median 90%, range = 0 - 100%, n = 69).

### Organization of an information search

#### Number of sources consulted

GPs who filled in a search logbook accessed up to three information sources, and on average 1.6 ± 0.8 sources (n = 71), to address their information need. Overall, 59% accessed only a single information source, 26% two sources, and 16% consulted three sources. Access to a second source was mostly prompted by an unsuccessful search in the first source (67% n = 27). Access to a third source information occurred (53.8% n = 13) after successfully consulting the second source and 46.2% of the time after failure consulting a second source.

#### Type of information sources consulted

GPs consulted colleagues (37.6%) most frequently, followed by books (22%), websites (15.6%) and search engines (11%); journals (2.7%) were marginal sources of information irrespective of source access order (Table 1). Respondents tended to access books more frequently as first than subsequent source, whereas electronic sources tended to be consulted more frequently as second (databases) or third (websites) sources. Consulting colleagues remained the preferred information source up to the 3rd source.

**Table 1:** Types and order of information sources consulted to address an information need

Type of information source	1^st ^source (n = 71)	2^nd ^source (n = 26)	3rd source (n = 12)	All sources (n = 109)
Colleague	36.6%	42.3%	33.3%	37.6%
Book	26.8%	15.4%	8.3%	22%
Website	12.7%	15.4%	33.3%	15.6%
Search engine	12.7%	7.7%	8.3%	11%
Database	2.8%	11.5%	8.3%	5.5%
Journal	2.8%	3.8%	0%	2.7%
Other	5.6%	3.8%	8.3%	5.5%

When consulted as first source, journals were associated with a more complex information need than other sources [single factor ANOVA, F (69,1) = 6.16, P < 0.05]; there was no evidence that consulting books was associated with less complex information needs [P = 0.12]. There was no evidence that consulting a colleague, website, database or search engine was influenced by the complexity of the information need (P > 0.27).

#### Time budget of information searching and decision rules used when successful and unsuccessful in an information source

**Figure 1:**
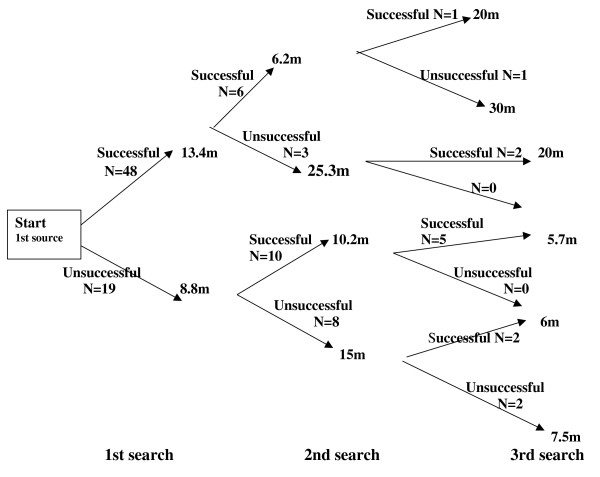
**Time profile of a search sequence**. N = number of GPs m = minutes.

Sixty-seven information search logbooks had complete data on searching time and success in each source accessed whether the searches were successful or not. Figure 1 shows a flowchart of the steps and decisions involved in these searches, with average search times indicated for each source sequentially accessed, and according to whether information searching was successful or unsuccessful in each source.

Out of 67 searches in the first source, 48 (71.6%) were successful and completed after an average of 13.4 minutes search time, and 19 (28.4%) were unsuccessful and completed after an average of 8.8 minutes search time (Table 2).

**Table 2:** Information searching time and decision to access a subsequent source according to source access order and search success in that information source

Source	Search time (min)		No of GPs (%) accessing subsequent source	
	Successful	Unsuccessful	Successful	Unsuccessful
First	13.4 ± 13.3 (1 - 60, n = 48)	8.8 ± 7.5 (2 - 25, n = 19)	N = 9 (18.8%)	N = 18 (94.7%)
Second	8.7 ± 5.2 (2 - 20, n = 16)	17.8 ± 16.1 (1 - 60, n = 12)	N = 7 (43.4%)	N = 7 (54.5%)
Third	11.1 ± 9.6 (2 - 30, n = 8)	11.8 ± 12.6 (2 - 30, 4)	N = 0 (0%)	N = 0 (0%)

Search times given as mean ± SD (range, sample size).

Thirty-nine (81.3%) of the 48 GPs successful in the first source stopped searching altogether, but nine (18.8%) went on searching in a second source. Six of those (66.7%) were again successful and left the second source after an average of 6.2 minutes. Three (33.3%) were unsuccessful and stopped searching after an average of 25.3 minutes, although this figure drops to 8.0 minutes when excluding an information search in a journal that lasted 60 minutes.

Out of the 19 GPs unsuccessful with the first source, only one (5.3%) stopped searching altogether and 18 (94.7%) went on searching in a second source. Ten of those (55.6%) were successful and left the second source after an average of 10.2 minutes, and 8 (44.4%) were again unsuccessful and left the second source after 15.0 minutes.

Nine (56.3%) out of 16 GPs successful in the second source stopped searching altogether, but seven (43.4%) went on searching another (third) source, and six (85.7%) of those were again successful in this source.

Out of 11 GPs unsuccessful in the second source, five (45.5%) stopped searching altogether, and six (54.5%) went on searching a third source. Four of those (66.7%) were successful in their third source.

Overall, 48 (75.0%) out of 64 GPs successful in one source stopped their search altogether, while 16 (25.0%) went on searching another source despite having found an answer. Out of 30 GPs that were unsuccessful in one source, six (20.0%) stopped their search altogether, and 24 (80.0%) accessed another source.

There was no evidence that GPs left their first information source more rapidly when unsuccessful than when successful in that source (mean of 8.8 min versus 13.4 min), (single factor ANOVA, [F(1,65) = 2.00, p = 0.16]). In contrast, the time spent searching in the second source was significantly longer when unsuccessful than when successful (mean of 17.8 min versus 8.7 min respectively; single factor ANOVA, [F(1,25) = 4.54, p = 0.04]). GPs spent a similar amount of time in the third source irrespective of whether successful or unsuccessful (mean 11.1 min versus 11.8 min respectively; single factor ANOVA, [F(1,10) = 0.009, p = 0.93]).

### Outcome of an information search

#### Search time

On average, GPs spent 12.1 minutes consulting an information source, and 17.7 minutes on a complete information search in up to three sources (16.2 minutes when successful, 30.3 minutes when unsuccessful,). Average search time per source depended significantly upon source type, ranging from 6.8 minutes when consulting books, to 11.8 minutes for colleagues, 12.5 minutes for search engines, 15.6 minutes for websites, and 22.0 minutes for journals and databases (Table 3).

**Table 3:** Search time, success and efficiency by type of information sources (all searches combined)

Information Source type	Search time (min)	Search success	Search efficiency (min search time needed per answer)
Book	6.8 ± 9.0 (1-45, n = 24)	70.8%	9.5
Colleague	11.8 ± 7.3 (2-30, n = 39)	74.4%	15.9
Search engine	12.5 ± 8.5 (3-30, n = 12)	50.0%	25.0
Website	15.6 ± 14.6 (1-60, n = 16)	56.3%	27.8
Journal	22.0 ± 32.9 (3-60, n = 3)	66.7%	33.0
Database	22.0 ± 15.5 (2-45, n = 6)	66.7%	34.3

Search times are given as expressed as mean ± SD (range, sample size).

#### Search success

Overall search success (finding an answer) was 69.7% in a single information source, and 89.4% at the end of a complete search. There was no difference in search success using different sources of information (Table 3).

#### Search efficiency

Average search efficiency (total search time in all sources combined, including unsuccessful search time, divided by the number of answers encountered in all sources) was 17.8 minutes per answer in any one source.

Information searching was most efficient when consulting books or colleagues, and least efficient when consulting databases or journals (Table 3).

## Discussion

This study is the first to apply OFT to understanding information searching behaviour among general practitioners and has revealed the search strategies undertaken when practicing under the time constraints seen in general practice. The results suggest that GPs try to achieve three main objectives when addressing an information need: minimizing the time taken to search for the relevant information, maximizing the chances of finding an answer, and maximizing the reliability of the information found. Application of foraging models has allowed new insights into the strategies and trade-offs used by GPs to achieve those often conflicting objectives. Application of OFT also indicates that, at the time the study was undertaken, possible gains in reliability from the use of electronic sources did not offset the optimising of time saved by consulting colleagues.

### Limitations

From the data it is not known whether or not the answers obtained by the GPs correspond to the best available evidence, as success was judged on the basis of the GPs' perception that their clinical question had been answered. The match between those answers and the best available evidence could be tested by submitting the same precise information questions to "perfect" medical information searchers. Such an approach has been applied to test the validity of diagnosis information retrieved from Google [18]. The study was based on a single population of primarily urban general practices in New Zealand. The Wellington GP population is heterogenous however and approximates overall to the demographic profile of New Zealand General Practitioners.

Family practice and primary care is a rapidly changing information and clinical evidence environment. Since the time this study was undertaken search speed and sophistication of internet search engines such as Gooogle continues to increase and anecdotally GP consultation use of the internet is increasing rapidly. The main objective of this study was to explore the analogy with optimal foraging theory and this is not dependent on the prioritising of foraging options at any particular point in time.

### Minimizing search time

GPs can potentially use two strategies to minimise information search times; either by consulting a single source and/or consulting sources that are less time-consuming to search or consult. GPs in this study often chose to access more than one source, and thus had to rely mainly on selecting less time consuming sources. Consulting colleagues and books took the shortest time; followed by consulting search engines and websites, while consulting journals and databases took the longest time. One of the main difficulties primary care physicians have previously reported when looking for electronic information is the amount of time it takes to do so [19-22]. The same barrier has been reported in relation to the use of computerised decision support systems [20] and computerised guidelines [21]. A recent study by Gravett et al [23] reports that colleagues are still a source of answers to immediate questions in the general practice.

### Maximizing chances to find a reliable answer

In accordance with OFT GPs appear to further optimise their search strategies by selecting more profitable information patches given that their success rate was highest when consulting colleagues, and lowest for websites and search engines. Nearly all GPs searched more than one source when unsuccessful, increasing search success from 69% when consulting a single source to 89% at the end of a complete search of up to three sources., But search success obviously depends also on the relevance of the information type to the information need, as suggested by the trend to consult books less frequently when faced with a complex information need. Complex needs were associated with consulting journals and increased search time, but not increased number of sources.

Many GPs who found an answer in one source went on to search an additional source. Although we have no data to explain this it seems likely that they do this to double check their newly found information. This is consistent with the importance that GPs attached to the reliability of information in their selection of information sources.

### Trade-offs between conflicting objectives

GPs face a conflict between maximizing search success (which increases when consulting more than one source) and minimizing search time (which also increases when consulting more than one source). Like animal foragers, GPs resolve this conflict by following a patch leaving rule (where a patch is an information source) aimed at minimizing the time wasted in an unsuccessful patch. Charnov's marginal value theorem predicts that a forager should leave a feeding patch ("giving-up time") if the rate of energy gain per unit time searched drops below the average rate of energy gain in all patches. If GPs follow this rule, they should on average spend 17.8 minutes in an information source before leaving when unsuccessful, which was indeed the case in their second sources. However, GPs unsuccessful in the first or third source left those sources much earlier than this (after 8.8 and 11.8 minutes respectively). This suggests that they leave these sources as soon as there are signs that the search may be unsuccessful (perhaps because there is no obvious information scent). When unsuccessful in the first source, they would have time to access another source; after a third source they presumably run out of time and/or information source options. When combining all sources irrespective of the order of access, the striking similarity between search time in any one source, whether successful (12.1 min) or unsuccessful (12.2 minutes), suggests that GPs' overall rule is the average time that it normally takes them to encounter an answer (the "information prey") when successful.

## Conclusions

While previous studies have shown that GPs consult mostly colleagues and books for their information needs, this is the first to provide quantitative data on the decision rules and rationale behind those choices. Based on optimal foraging theory, GPs would predictably consult colleagues and books for their information needs because those sources were, at the time of the study, the most efficient at providing an answer per unit time searched. In this regard they were following a process of 'grazing' for information which combines to a varying extent clinical experience with access to information sources that had a degree of evidence base [24]. It seems unlikely that GPs will use EBM associated information sources until their profitability can be dramatically increased to match the time constraints of real world general practice. Continuous improvements in data retrieval systems as Google and Google Scholar [25] suggest that formal information databases will be unlikely to be used in routine clinical practice except for occasional complex searching, but will be reserved for research and guideline development. Clinical practice is clearly in transition with the designers of electronic information sources within General Practice seeking products that will at the very least match the information reward of consulting colleagues, and more specifically products that will provide an answer quickly. New clinical support tools such as ' up to date' (http://www.uptodate.com) or the BMJ's 'clinical evidence' (http://clinicalevidence.bmj.com) have greater clinical relevance and are much faster than previous generation products. It remains to be seen how their overall face validity and reliability for many practitioners compares to asking colleagues for an informed opinion.

The foraging model developed in this study could also be used to predict information search strategies under hypothetical source profitabilities. Such modeling would also enable analysis of the sensitivity of information search outcomes to changes in various parameters, and differing levels of constraint or support. It is hoped that these results will improve the recognition of the GPs trade-offs between conflicting objectives in addressing their information needs (e.g. minimizing search time, maximizing chances of finding a reliable answer.

## Abbreviations

GPs: General Practitioners; OFT: Optimal Foraging Theory; EBM: Evidence-based Medicine.

## Competing interests statement

The authors declare that they have no competing interests.

## Authors' contributions

This study was part of a PhD thesis by MD on "The Application of the Foraging Theory to the Management of Information by General Practitioners". MD conceived the original idea for the study, contributed to the design, undertook the analysis of data and wrote the first draft of the manuscript. AD was primary supervisor to MD, contributed to the design and analysis of data and undertook editing of final drafts of the manuscript. JCS contributed to the design of the study, provided expertise regarding foraging theory, was secondary supervisor to MD and reviewed drafts of the manuscript. All authors read and approved the final manuscript.

## Pre-publication history

The pre-publication history for this paper can be accessed here:

http://www.biomedcentral.com/1471-2296/12/90/prepub

## Supplementary Material

Additional file 1 **Appendix 1. Clinical information log**. The clinical information log used by the GPs to collect information about their search strategies. Click here for file
