# Supplementary material for: The application of foraging theory to the information searching behaviour of general practitioners
Source: BMC Fam Pract. 2011 Aug 23;12:90. doi: 10.1186/1471-2296-12-90 (PMC3175159; doi:10.1186/1471-2296-12-90)
Supplement: Additional file 1 — Appendix 1. Clinical information log. The clinical information log used by the GPs to collect information about their search strategies. [file 1471-2296-12-90-S1.DOC]

## Appendix 1 : CLINICAL INFORMATION SEARCH LOG

**[ B ]**

| **9.1. Time when information need raised:**  Date: Hour: Min: |
| --- |
| **9.2. Was this during a consultation with a patient?** *(Tick one)*  [ ] Yes [ ] No |
| **9.3. Please provide a summary of your information question:** |
| **9.4. How important is it, for the patient’s care, to get this information** (*Tick one*):  [ ] No importance [ ] Low importance [ ] Moderate importance [ ] High importance |
| **9.5. When do you need this information:** *(specify number)*  Within: ………. Mins; ………. Hrs; ………. Days; ………. Weeks |
| **9.6. Estimated time required to find this information:** |
| **9.7. Maximum time that you can allocate to find the information:** |
| **9.8. Estimated likelihood of finding the information within the desired time (%):**  **10. Number of information sources consulted** |

| **1^st^ INFORMATION SOURCE CONSULTED – Source descriptors** |
| --- |
| **10.1. Information source** *(Tick one):*  **Verbal information source**  [1] Colleague (Speciality and location e.g. same practice, same city, within NZ, or overseas)  …………………………………………………………………………………………  **Published/electronic information sources**  [ 2 ] Book (Reference name, author, **year** ……………………………………………………)  [ 3 ] Journal (Name, issue………………………………………………………………………)  [4 ] Website (URL address………………………………………………….…………………)    [5 ] Database (Name…………………………………………………………………… …...)  [6 ] Search engine (Name………………..URL address found………………………………..)    [7 ] Other (specify ……………………………………………………………………………) |
| 10.2. Where did you access the source (*Tick one*): [ ] Home [ ] Office [ ] Clinic/Practice? [ ] Library  [ ] Other (specify) |
| **10.3. Estimated time spent to access the source:** …………………………………… |
| 10.4. Reasons for choosing this source (*Tick as many as apply*): [ a ] Relevance [c ] Availability [e ] Ease of access [ g ] Cost  [b ] Familiarity [d ] Easy to search [ f] Previous success [h ] Anticipated success  **10.5. Provides information items that is** (*Tick as many as apply*):  [a ] Up-to-date [c] Reliable [d] Understandable  [b ] Synthesized (e.g. Reviews) [e] Immediately applicable    [f] Other (specify): ………………………………………………………………… |
| **10.6. Previous searching of this source** (*Tick one***):**  [1] Never [2 ] Rarely [3 ] Sometimes [4] Often |
| **10.7. How did you find this information?** *(Tick one)*  [1] Search engine [3] Stored from previous search [5] Librarian  [2 ] Link or Reference [4] Colleague [6] Other……………... |
| **10.8. Previous success rate in this source** (*estimated %): ………………………* |
| **1^ST^ INFORMATION SOURCE CONSULTED – Search descriptors** |
| **11.1. Search start time:**  Date: Hour: Min: |
| **11.2.a *If verbal*** information source ***[colleague]*** consulted, *please answer here* **(*if not go to 3***)  **[Sequence of events]** (*Tick one*)  [1 ]I explained my information need, and obtained the needed answer;  [2]The answer was not exactly what I needed. I had to clarify the information need again;  [3]I had to clarify the information need for a third time to get the needed answer;  [4]I had to search another information source to obtain the answer;  [5]Other (specify):…………………………………. |
| *11.3.a If published/electronic* information source consulted*, please answer here (if not go to 4):*Criteria (search strategy) used for seeking information items in this source(*tick as much as you used):* [1] Exact topic [4] Relevant topics [7] Relevant authors  [2] Relevant journals [5] Relevant methods [8] Publication date  [3] Keywords [6] Subject heading [9] Index  [10] Other (specify)………… …………….. 11.3.b. Browsing method used for first selection of relevant information items: (*Tick as many as apply)* [1] Title [3] Abstract [4] Author [5] Heading/table of content  [2] Index  [6] Other (specify):……………………………………………. |
| 11.4. If searching for your information *electronically*, please time your search and fill out this question as you proceed. (*if not go to Question 5)* **Total number of items found in this source to start with**…………………….   1. *As you browse the titles,* **how many items have you viewed** after **2 mins? [1]**…….., and **how many have you chosen as relevant? [2]…………**      1. *If you continue,(if* ***not*** *go toQ****5****),* **how many items have you viewed during the next 2 mins? [1]……… and how many have you chosen as relevant? [2]………...**      1. *If you continue, (if* ***not*** *go to Q****5****),* **how many items have you viewed during the next 2 mins? [1]… and how many have you chosen as relevant? [2]……...** |
| **11.5. Search (retrieval) stopping time** (*on this source*):  Date: Hour: Min: |
| **11.6. Estimated time to find the information** (*excluding filling the log*) ………………… |
| 11.7.a *If published information* source consulted, *please answer here (if not go to 8):*What did you read? *(Tick one)* [1] The full text [2] The abstract / summary [3] Other …………………… 11.7.b Estimated time needed to read and critically appraise the retrieved information items: …………………. |
| **11.8. Why did you stop searching this source now:** (*Tick as many as apply*)  [a] I found the right answer/enough information.  [b] Would take me too long to find answer in this source.  [c] I don’t think the answer is there.  [d] I try another source that may be better.  [e] Source too busy (e.g., colleague).  [f] Unable to finish now, will return to source later.  [g Time elapsed for this source.  [h] No more time for this search.  [i] I don’t think the answer exists.  [j] Other (specify) …………………………………………………………………. |
| **11.9. Please provide a short summary of the answers found in this source:** |
| **11.10. What will you do next: (***Tick one***)**  [1] Apply search result [3] Try other source immediately  [2] Try other source later [4]Ask somebody to do it for me  [5] Other (specify) ………………………………………………………………… |
| **11.11.a Will you stop searching this information need altogether here:**  [ ] Yes [ ] No |
| **11.12. If so, what are the reasons** (*Tick one or more*):  [a] Found enough information [c] Answer probably doesn’t exist  [b] Cannot allocate more time [d] Search budget expended  [e] Other (specify): …………………………………………………… |
| **11.13. Information found so far** *(Tick one*)  [1] Changed my practice  [2] Confirmed my knowledge  [3] Did not answer my question  [4] Other (specify): ………………………………………………………….. |

***ONLY If you continue the searching using further information sources, please continue filling out the Search Log for the next information sources. Otherwise stop here.***

| **2^nd^ INFORMATION SOURCE CONSULTED – Source descriptors** |
| --- |
| **12.1.a Information source** *(Tick one):*  **Verbal information source**  [1] Colleague (Speciality and location e.g. same practice, same city, within NZ, or overseas)  …………………………………………………………………………………………………..  **Published/electronic information sources**  [2] Book (Reference name, author, **year** ……………………………………………………)  [3] Journal (Name, issue………………………………………………………………………)  [4] Website (URL address………………………………………………….…………………)    [5] Database (Name……………………………………………… …...)  [6] Search engine (Name………………..URL address found………………………………..)    [7] Other (specify ……………………………………………………………………………) |
| 12.2. Where did you access the source (*Tick one*): [1] Home [2] Office [3] Clinic/Practice? [4] Library  [5] Other (specify) |
| **12.3. Estimated time spent to access the source:** …………………………………… |
| 12.4. Reasons for choosing this source (*Tick as many as apply*): [a] Relevance [c] Availability [e] Ease of access [g] Cost  [b] Familiarity [d] Easy to search [f] Previous success [h ] Anticipated success  **12.5. Provides information items that is** (*Tick as many as apply*):  [a] Up-to-date [c Reliable [d] Understandable  [b] Synthesized (e.g. Reviews) [e] Immediately applicable    [f] Other (specify): ………………………………………………………………… |
| **12.6. Previous searching of this source** (*Tick one***):**  [1] Never [2] Rarely [3] Sometimes [4] Often |
| **12.7. How did you find this information?** *(Tick one)*  [1] Search engine [3] Stored from previous search [5] Librarian  [2] Link or Reference [4] Colleague [6] Other…… |
| **12.8. Previous success rate in this source** (*estimated %): ………………………* |

| **2^nd^ INFORMATION SOURCE CONSULTED – Search descriptors** |
| --- |
| **13. Search start time:**  Date: Hour: Min: |
| **13.1. *If verbal*** information source ***[colleague]*** consulted, *please answer here* **(*if not go to 3***)  **13.2 [Sequence of events]** (*Tick one*)  [1]I explained my information need, and obtained the needed answer;  [2]The answer was not exactly what I needed. I had to clarify the information need again;  [3]I had to clarify the information need for a third time to get the needed answer;  [4]I had to search another information source to obtain the answer;  [5]Other (specify):…………………………………. |
| *If published/electronic* information source consulted*, please answer here (if not go to 4):*13.3.a Criteria (search strategy) used for seeking information items in this source(*tick as much as you used):* [1] Exact topic [4] Relevant topics [7] Relevant authors  [2] Relevant journals [5] Relevant methods [8] Publication date  [3] Keywords [6] Subject heading [9] Index  [10] Other (specify)………… …………….. 13.3.b Browsing method used for first selection of relevant information items: (*Tick as many as apply)* [1] Title [3] Abstract [4] Author [5] Heading/table of content  [2 ] Index  [6] Other (specify):……………………………………………. |
| If searching for your information electronically, please time your search and fill out this question as you proceed. (*if not go to Question 5)* **13.4.** **Total number of items found in this source to start with**…………………….  **a***. As you browse the titles,* **how many items have you viewed** after **2 mins?** ……………, and **how many have you chosen as relevant? …………………**    **b.** *If you continue,(if* ***not*** *go toQ****5****),* **how many items have you viewed during the next 2 mins? ……… and how many have you chosen as relevant? ……...**     1. *If you continue, (if* ***not*** *go to Q****5****),* **how many items have you viewed during the next 2 mins? ……… and how many have you chosen as relevant? ……...** |
| **13.5 Search (retrieval) stopping time** (*on this source*):  Date: Hour: Min: |
| **13.6 Estimated time to find the information** (*excluding filling the log*) ………………… |
| 13.7.a *If published information* source consulted, *please answer here (if not go to 8):*What did you read? *(Tick one)* [1] The full text [2] The abstract / summary [3] Other …………………… 13.7.b Estimated time needed to read and critically appraise the retrieved information items: …………………. |
| **13.8. Why did you stop searching this source now:** (*Tick as many as apply*)  [a ] I found the right answer/enough information.  [b ] Would take me too long to find answer in this source.  [c] I don’t think the answer is there.  [d] I try another source that may be better.  [e] Source too busy (e.g., colleague).  [f] Unable to finish now, will return to source later.  [g] Time elapsed for this source.  [h] No more time for this search.  [i] I don’t think the answer exists.  [j] Other (specify) …………………………………………………………………. |
| **13.9 Please provide a short summary of the answers found in this source:** |
| **13.10. What will you do next: (***Tick one***)**  [1] Apply search result [3] Try other source immediately  [2] Try other source later [4] Ask somebody to do it for me  [5] Other (specify) ………………………………………………………………… |
| **13.11. Will you stop searching this information need altogether here:**  [ ] Yes [ ] No |
| **13.12 If so, what are the reasons** (*Tick one or more*):  [a] Found enough information [c] Answer probably doesn’t exist  [b] Cannot allocate more time [d] Search budget expended  [e] Other (specify): …………………………………………………… |
| **13.13. Information found so far** *(Tick one*)  [1] Changed my practice  [2] Confirmed my knowledge  [3] Did not answer my question  [4] Other (specify): ………………………………………………………….. |

***ONLY If you continue the searching using further information sources, please continue filling out the Search Log for the next information sources. Otherwise stop here.***

| **3^rd^ INFORMATION SOURCE CONSULTED – Source descriptors** |
| --- |
| **14.1.a Information source** *(Tick one):*  [1] Colleague (Speciality and location e.g. same practice, same city, within NZ, or overseas)  …………………………………………………………………………………………………..  [2 ] Book (Reference name, author, **year** …………………………………………………)  [3 ] Journal (Name, issue……………………………………………………………………)  [4 ] Website (URL address……………………………………………….…………………)    [5 ] Database (Name………………………………………………………………… …...)  [6 ] Search engine (Name………………..URL address found……………………………..)    [7 ] Other (specify …………………………………………………………………………) |
| 14.2 Where did you access the source (*Tick one*): [1] Home [2 ] Office [3 ] Clinic/Practice? [4 ] Library  [5 ] Other (specify) |
| **14.3 Estimated time spent to access the source:** …………………………………… |
| 14.4. Reasons for choosing this source (*Tick as many as apply*): [a ] Relevance [c ] Availability [e ] Ease of access [g ] Cost  [b ] Familiarity [d ] Easy to search [f ] Previous success [h ] Anticipated success  **14.5 Provides information items that is** (*Tick as many as apply*):  [a ] Up-to-date [c] Reliable [d ] Understandable  [b] Synthesized (e.g. Reviews) [e] Immediately applicable    [f] Other (specify): ………………………………………………………………… |
| **14.6 Previous searching of this source** (*Tick one***):**    [1 ] Never [2 ] Rarely [3] Sometimes [4 ] Often |
| **14.7 How did you find this information source?** *(Tick one)*  [1 ] Search engine [2 ] Stored from previous search [3 ] Librarian  [4 ] Link or Reference [ 5] Colleague [6 ] Other……………... |
| **14.8 Previous success rate in this source** (*estimated %): ………………………* |
| **3^rd^ INFORMATION SOURCE CONSULTED – Search descriptors** |
| **15. Search start time:**  Date:……………….Hour:……………………Min:………………………….. |
| ***15.2 Answer this question ONLY If [colleague]*** consulted, *otherwise* ***go to Q 3***)  **[Sequence of events]** (*Tick one*)  [1 ]I explained my information need, and obtained the needed answer;  [2 ]The answer was not exactly what I needed. I had to clarify the information need again;  [3 ]I had to clarify the information need for a third time to get the needed answer;  [4 ]I had to search another information source to obtain the answer;  [5 ]Other (specify):…………………………………. |
| *15.3.a. If published/electronic* information source consulted*, please answer here (if not go to 4):*Criteria (search strategy) used for seeking information items in this source(*tick as much as you used):* [1 ] Exact topic [4 ] Relevant topics [7] Relevant authors  [2 ] Relevant journals [5] Relevant methods [8] Publication date  [3 ] Keywords [6] Subject heading [9] Index  [10 ] Other (specify)………… …………….. 15.3.b. Browsing method used for first selection of relevant information items: (*Tick as many as apply)* [1] Title [3] Abstract [4] Author [5] Heading/table of content  [2] Index  [6] Other (specify):……………………………………………. |
| 15.4. *ONLY* If searching for your information electronically, please time your search and fill out this question as you proceed. (*if not go to Question 5)* **Total number of items found in this source to start with a**…………………….   1. *As you browse the titles,* **how many items have you viewed** after **2 mins? 1.**……………, and **how many have you chosen as relevant? 2. …………………**     **c*.*** *If you continue,(if* ***not*** *go toQ****5****),* **how many items have you viewed during the next 2 mins? 1……… and how many have you chosen as relevant? 2……...**     1. *If you continue, (if* ***not*** *go to Q****5****),* **how many items have you viewed during the next 2 mins? 1……… and how many have you chosen as relevant? 2……...** |
| **15.5.Time when you ended searching this source**:  Date: …………………Hour: …………………….Min:…………………………….. |
| **15.6. Estimated time to find the information** (*excluding filling the log*) ………………… |
| 15.7.a ANSWER this question ONLY *If published* source consulted, otherwise *go to Q 8):*What did you read? *(Tick one)* [1 ] The full text [ 2] The abstract / summary [3 ] Other …………………… 15.7.b Estimated time needed to read and critically appraise the retrieved information items: **………………………………………………….** |
| **15.8. Why did you stop searching this SOURCE now:** (*Tick as many as apply*)  [a] I found the right answer/enough information.  [b] Would take me too long to find answer in this source.  [c] I don’t think the answer is there.  [d] I try another source that may be better.  [e] Source too busy (e.g., colleague).  [f] Unable to finish now, will return to source later.  [g] Time elapsed for this source.  [h] No more time for this search.  [i] I don’t think the answer exists.  [j] Other (specify) …………………………………………………………………. |
| **15.9 Please provide a short summary of the answers found in this source:** |
| **15.10 What will you do next: (***Tick one***)**  [1 ] Apply search result [ 3] Try other source immediately  [2 ] Try other source later [4] Ask somebody to do it for me  [5 ] Other (specify) ………………………………………………………………… |
| **15.11 Will you stop searching this information NEED altogether here:**  [1 ] Yes [2 ] No |
| **15.12 If so, what are the reasons** (*Tick one or more*):  [a] Found enough information [c] Answer probably doesn’t exist  [b] Cannot allocate more time [d] Search budget expended  [e] Other (specify): …………………………………………………… |
| **15.13. Information found so far** *(Tick one*)  [1 ] Changed my practice  [2 ] Confirmed my knowledge  [3 ] Did not answer my question  [4 ] Other (specify): ………………………………………………………….. |
